# Repertoire Construction for Critical Cross-Cultural Literacy of English Majors: Based on the Research Paradigm of Systemic Functional Linguistics

**DOI:** 10.3389/fpsyg.2022.906175

**Published:** 2022-06-27

**Authors:** Ran Zhao, Danyun Lu

**Affiliations:** National University of Defense Technology, Changsha, China

**Keywords:** critical cross-cultural literacy, individual identity, repertoire, systemic functional linguistics, cultural globalization

## Abstract

The ambiguous development trend of cultural globalization brings both opportunities and challenges to China’s cultural development. English major in colleges and universities, a discipline of cross-cultural education, should look at the cultural communication of the target country dialectically based on the national consciousness of the home country. Since the end of the 20th century, administrators and scholars have paid attention to critical thinking, critical cultural awareness, and critical skills in cross-cultural communication, which are important components of the cross-cultural meaning system. Therefore, all these are collectively referred to as critical cross-cultural literacy (CCCL). On the basis of the research paradigm of systemic functional linguistics (SFL), a language is a semiotic system that creates meaning. Thus, to help students construct and improve their individual CCCL repertoire, teachers need to guide them to critically study and analyze the discourse purpose of the textbook author as well as their language methods and strategies to enrich their meaning potential.

## Introduction

The future development trend of cultural globalization is still unclear. Whether the world cultural pattern in the future is prone to western cultural hegemony, multicultural coexistence, or the rise of Asian culture has not been determined in academic circles (Tomlinson, 1999; Liu, 2018; Manathunga, 2018; Wei, 2018) and whether China can seize the development opportunities in the challenge of cultural globalization have become the focus of China’s cultural development and the key to cultivate talents in higher education. Most of the textbooks used by contemporary Chinese undergraduates in compulsory education and senior high school are English originals or English stories. The teaching goal is to master vocabulary and grammar and it rarely involves the cultivation of critical thinking. However, when they grow up and study at the university, they cannot ignore the significance of thinking. English major, a key discipline in cross-cultural education, should not only concentrate on the national consciousness of the home country but also critically explore the national consciousness of the target country implied in cross-cultural communication, thereby promoting the national cultural development strategy from the perspective of professional talent cultivation. Therefore, on the basis of consolidating language skills, its teaching goal is to focus on logical thinking, critical thinking, cross-cultural literacy, etc. These changes require us to update our teaching methodology.

All words are meaningful. Since the 1990s, linguists began to realize the importance of language for cross-cultural education (Byram, 1997; Kramsch and Zhu, 2016). On the one hand, language makes meaning in the context of culture, so it has explicit national consciousness. On the other hand, as language is a tool of persuasion and communication, national consciousness is implicit in it. Therefore, it is necessary for the students in English majors to recognize these explicit and implicit meanings contained in the language in cross-cultural communication. The educational objectives related to national consciousness identification in cross-cultural communication were defined as Critical Cross-cultural Literacy (CCCL). Empirical research shows that students in English majors are short of CCCL. Accordingly, the final goal of the paper is to construct students’ repertoire of CCCL, that is, to realize CCCL in language based on their various identities.

## Literature Review and Theoretical Framework

### Connotations of Critical Cross-Cultural Literacy

At the end of the 20th century, with the intensification of globalization, the opportunities and difficulties faced by countries all over the world in cross-cultural communication brought a new element to cross-cultural research—critical literacy, which usually involves “critical cultural awareness” (CCA), “critical thinking,” and “evaluative skills.” Byram focused on the significance of language in cross-cultural communication. He integrates linguistics and intercultural communication together to form a model of “Intercultural Communicative Competence,” the core element of which is CCA, also known as political education. CCA is “an ability to evaluate critically and on the basis of explicit criteria, perspectives, practices, and products in one’s own and other cultures and countries”. Its basis is evaluation (Byram, 1997, 2009). After entering the 21st century, Deardorff, together with many administrators and cross-cultural research scholars, participated in the Delphi study. The specific skills through scholars’ consensus are skills to “analyze, interpret, and relate,” which relate to the critical abilities. Based on the Delphi study, Deardorff constructed the “Pyramid Model of Intercultural Competence,” in which requisite attitude, the most critical element of the model, is located in the basic position (Deardorff, 2006). Gao proposed a conceptual framework consisting of two mutually interactive dimensions of knowing and doing, which is used for assessing Chinese college students’ intercultural communication competence. The knowing dimension is about the value, while the doing dimension refers to the intercultural behaviors based on the value. The former consists of knowledge, consciousness, and critical thinking. Critical thinking refers to “pure theoretical and conceptual thinking by using logical reasoning” and constitutes a logical system in cross-cultural communication. The latter consists of attitude, skills, and strategies. Attitude including openness, inclusiveness, and flexibility refers to “people’s tendency to evaluate people or things under the control of certain cultural values” (Gao, 2014). In both knowing and doing dimensions, critical thinking and evaluative abilities are included.

Owing to the needs of China’s national education development, the interpretation of cross-cultural competence in the “National Standard for Teaching Quality of Foreign Language and Literature” (referred to as the “Standard”) and the “Teaching Guide for Undergraduate Foreign Language and Literature Majors in Colleges and Universities (Volume I)–Teaching Guide for English Majors” (referred to as the “Guide”) issued in recent years is related to three independent literacies, namely, “cross-cultural awareness,” “critical thinking,” and “dialectical thinking,” which are all necessary literacies for cross-cultural communication.

Although it is not uniformly named, administrators and scholars have paid attention to the importance of analytical and evaluative literacy in cross-cultural communication. However, diverse names and scattered components are not easy to form thought-directed classroom teaching goals. Therefore, this study summarizes them with CCCL, which refers to the critical literacy in cross-cultural communication, including analytical ability, evaluative skills, critical ability, and other related literacy. It is not only the integration of cross-cultural learning and national consciousness but also an important literacy goal for foreign language courses in high school to realize the integration of knowledge, ability, and ideological and political education.

### Literature Review of Pedagogical Practices on Critical Cross-Cultural Literacy

In recent years, scholars have devoted themselves into investigating the pedagogical practice of the competencies related to CCCL, with some majoring in cross-national education. For instance, Jackson centers on the course “Intercultural Transitions: Making Sense of International Experience” to help students better understand their intercultural experiences. The participants required include either students with overseas experience or students from other countries. Students share their international experiences in the class, and through structured reflection, they gradually integrate the critical elements into their discussions and essays (Jackson, 2015). Yulita used Skype to make students from Argentina and United Kingdom work together to design a leaflet about a real political issue. One of the goals of this project is to foster the ability of critical analysis, which is developed in students’ choice of image for the leaflet through the discussion on Skype (Yulita, 2017). Others prefer to do domestic teaching practice. For example, in the light of experiential learning strategies, Durgun combined the course “Narratives of Identity” with “Human Creativity: Science and Art” to foster their cultural-related abilities, such as critical observation, conceptualization of problems and solutions, and reflection. He utilizes the experiences and feedback from his peers and educators to finish the individual and group projects. The final exhibition form is drawing, painting, and so on (Durgun, 2019). Aksikas shared the teaching experience of a methodology course in cultural studies, the goal of which is critical and self-reflexive. Therefore, teaching steps can be divided into two parts: introduce methodologies of cultural studies and encourage students to complete some research projects. In the second step, short lectures as well as group work discussions and activities are structured (Aksikas, 2019).

These teaching practices are mainly about the cultivation of evaluation and reflection, but their teaching activities are group work or peer reviews, which ignores the importance of individual development. Some scholars propose that culture is a process of meaning-making, thus English language teaching should be applied from the perspective of discourse. Cultures communicate with each other by means of individual members of the cultural community. The discourse systems are multiple, and sometimes contradict each other as reflecting the multiple identities that people produce through interactions (Scollon and Scollon, 2001; Kramsch and Zhu, 2016). Thus, we propose to have a study on students’ CCCL repertoire from the perspective of discourse.

### Research Paradigm of Systemic Functional Linguistics

Aiming to solve the problem of discourse generation or meaning-making, systemic functional linguistics (SFL) with “appliable” is a linguistic theory with a dialectical critical view for developing CCCL. As for SFL, its insight into the laws of human language development, individual language development, and text construction fully display Marxist dialectical materialism viewpoint and language viewpoint (Halliday et al., 2015; Hu, 2016). SFL regards language as a semiotic system, which creates meaning. It constructs a systematic theoretical framework for describing, communicating, and organizing languages in the context of culture. In this theory, human experience activities, language organization, and interpersonal interactions are summarized into ideational, textual, and interpersonal functions (Halliday and Matthiessen, 2014). The interpersonal function is the meaning potential for the speaker who acts as an intruder and tries to show the attitude and evaluation through language. The meaning potential is what learners need to build up in language education. It is a resource for creating meaning. So first of all, students need to construe a linguistic system, which is instantiated in the form of text and can be drawn on in reading. Then, they need to learn how to use it (Halliday, 1991). What teachers need to do is guide students to recognize how language works in the context of culture and help them to construct their own meaning potential.

Language systems are organized by strata, rank, and metafunction on a hierarchy referred to as realization (Martin, 2009; Gebhard et al., 2013, 2014), which means that culture is realized in language; meanings are realized as wordings; wordings are realized as writing or sounds. The appraisal system meets the need of context for evaluation and effectuates the alliance or betrayal between speakers and listeners/readers in thought and viewpoint *via* the evaluation of people’s emotions and behaviors as well as the values of things, which are all realized in wordings. So, students can use it to construe language strategies in the reading material.

Attitude, graduation, and engagement (three subsystems) of this theory correspond to attitude type, attitude strength, and viewpoint source of discourse evaluation, respectively (Martin and White, 2005). To be specific, attitude includes three subsystems: effect, judgment, and appreciation. All these subsystems share both positive and negative evaluations, and they respectively, represent the evaluations of speakers’ emotions, behaviors, and the composition and value of the text/process/phenomenon. As for effect, it is divided into disinclination/inclination, unhappiness/happiness, in security/security and dissatisfaction/satisfaction. The first one is the irrealis effect, representing the desire for reality, while the latter three are the realis effect, referring to the response to reality. The judgment explains our attitudes toward people and their behaviors, including two types of standards: social esteem and social sanction. Specifically, the former is about normality, capacity, and tenacity and the latter is about veracity and propriety. The appreciation system deconstructs the evaluation of things, which can be divided into three types: reaction, composition, and valuation. In addition, graduation (one of the subsystems) focuses on the intensity of attitude expression which is reflected by force and focus. The force refers to different intensities of languages while focus can be divided into two types, namely sharpen and soften. Speakers adjust the intensity of speech by changing the force and focus. The intervention system involves monogloss and heterogloss. The former represents the discourse viewpoint from the speaker while the latter represents that from others (except the speaker). Therefore, this evaluation method is related to whether the speaker takes the discourse responsibility.

As we have said, a language is a semiotic system that creates meaning, so it is also called a semogenic system. It owns three dimensions, one of which is ontogenesis. Ontogenesis focuses on the development of repertoires of language users. In terms of the concepts of reservoir and repertoire proposed by Bernstein, individuals interact in the reservoir to form a system and determine the affiliation and persona through the individual coding orientation to establish a repertoire, thus completing the process of “mass” → “pair” → “person” (Martin, 2006; Zhu, 2012). Therefore, individuation is a theory depicting the relationship between individuals and social culture and the development of individual meaning potential on the basis of the research paradigm of SFL, and it also reveals the differences among language users. Individuation concentrates on how individuals make choices in reservoir, complete identity construction, and establish a repertoire, namely, individual literacy. Accordingly, on the basis of SFL’s language philosophy and semantic system, the paths to improve individual CCCL repertoire of English majors in colleges and universities were the research object, and the appraisal system was the theoretical framework in this research.

## Research Design

This research consisted of two parts: A questionnaire survey and a language test. The questionnaire survey has its advantages in avoiding interpersonal interference resulting from interviews, taking more respondents as samples, and applying written answers to open questions to discourse analysis. Besides, the language tests were conducted to make up for the deficiency that the respondents’ evaluation of their own behavior abilities reflected in the questionnaires that were often subjective and uncertain.

In accordance with the literacy contained in CCCL and Facione’s investigation of critical thinking (Facione, 2020), closed, semi-closed, and open questions for cross-cultural knowledge, consciousness, evaluation, and emotional qualities were set in the questionnaires. The language test composed of two reading comprehension articles (five single-choice questions and two essay questions) was set to examine students’ evaluation ability. After the questionnaire and the test paper were prepared, a total of 30 respondents took the pre-test to ensure the validity, and adjustments were made based on the test results. The students participating in this questionnaire and language test were all English majors in a “double first-class” university in China. A total of 25 students, including sophomores, juniors, and seniors, were randomly selected by the researchers to take a one-and-a-half-hour collective test in the same classroom. In this research, 75 questionnaires and language test papers were given out first. Finally, all those given out were collected with an effective recovery rate of 100%.

**FIGURE 1:**
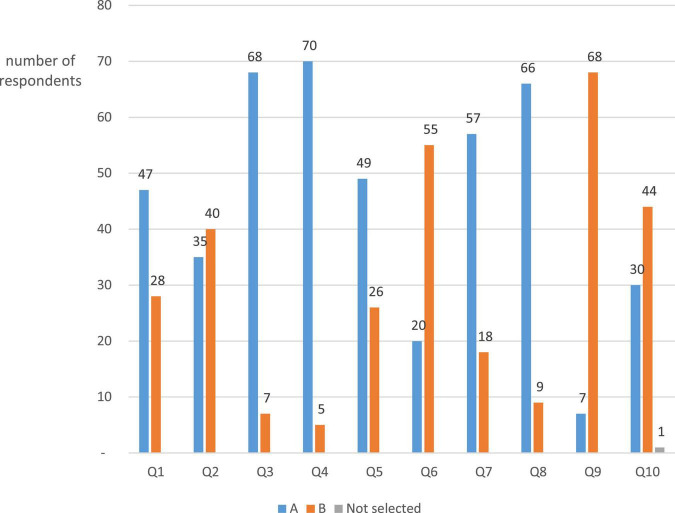
Data statistics of self-test questions in the questionnaire.

According to the background information of respondents participating in this questionnaire survey, there were 44 students whose NCEE English scores were within the first 10% of total scores, 31 students within the first 10–20%, and no students lower than the first 20%. The survey demonstrated that 96% of the students could answer the questions related to cross-cultural knowledge, so they had cross-cultural knowledge. Besides, 72% of the students had cross-cultural awareness while the cross-cultural awareness was weak among 28%. A total of 21.3% of the students correctly answered all the questions related to evaluation ability, 53.3% had an accuracy of 50%, and 25.3% gave wrong answers to all questions (see Table 1). According to Facione’s investigation (Facione, 2020), students with critical dispositions tended to choose option A in self-test questions 1, 3, 4, 7, and 8, and option B in questions 2, 5, 6, 9, and 10. As seen in Figure 1, it was difficult for a large number of students to develop perfect critical dispositions even in self-tests. The NCEE English scores of the students (“good at English”) who took the test were higher than 120 scores (national version) or 96 scores (Jiangsu version), and the knowledge-related parts of the questionnaire survey and the language test also proved that these respondents were traditionally “good students”, but they still had the problem of low accuracy in evaluation questions and self-test personality. In other words, they had insufficient evaluation literacy or critical literacy.

**TABLE 1:** Background information of respondents and test results.

Number of people in each grade			English score of national college entrance examination (NCEE)			Accuracy of evaluation questions		
		
Sophomore	Junior	Senior	10%	10–20%	20–30%	100%	50%	0
25	25	25	44	31	0	21.3%	53.3%	25.3%

*The total English scores in NCEE (Jiangsu version) are 120, and those in NCEE (national version) are 150. In this research, therefore, scores were expressed by percentages. NCEE (national version): 10% (scores: 135), 20% (120), and 30% (105). NCEE (Jiangsu version): 10% (108), 20% (96), and 30% (84).*

For this reason, English teaching should strengthen the cultivation of critical literacy and enrich students’ repertoires on the premise of maintaining high-quality cultural knowledge teaching. In 2022, a new version of the English curriculum standard for compulsory education was released. Compared to the old one, the new version pays more attention to the logical analysis of reading comprehension. For instance, it requires students to understand the implicit and explicit meanings in the article, evaluate the author’s attitude, and have a deep understanding of the articles’ structure. All these requirements are just released so the reform is for the new generation. Then, how about the students who grow up with the old version standard? The universities, especially the English major, should take the responsibility to foster their CCCL. Students in the English major have skills in listening, speaking, reading, and writing. They can read some foreign language articles by themselves and do not need translated materials. Some views implicated in the article may affect students’ ideas. Therefore, teaching students the way to evaluate the reading material can help them to improve their CCCL.

## Improvement of Individual Critical Cross-Cultural Literacy Repertoire

Classroom discourse analysis refers to the analysis of texts in the classroom context, including the teacher–student dialogues and teaching materials (Hammond, 2011; Huang, 2020). As the main discourse mode of school education, classroom discourse has become the focus of CCCL education research. Speakers’ words are purposeful, namely, aiming to establish and maintain appropriate social relations with listeners/readers by giving or asking for information/services (Halliday and Matthiessen, 2014). Language use involves two dimensions: receptive and productive. In terms of interpersonal communication, language use refers to a two-way activity of “input” and “expression” (Saville-Troike, 2019). English functions as an educational language in classroom discourse. To help the students construct and improve their individual CCCL repertoire, teachers need to guide them to critically study and analyze the discourse purpose of the textbook author as well as their language methods and strategies to enrich their meaning potential. After that, students should develop their own language strategies and styles by choosing coding orientations in classroom activities. At last, they transform from listeners/readers to speakers.

### Critical Analysis Path of Coding Orientation in Classroom Discourse

Individuation of SFL focuses on the relationship between identity recognition and language coding, involving a two-way process of individuation and affiliation. The former refers to the process where semiotic resources are allocated to different individuals to construct their identities, while the latter represents a process in which individuals utilize their resources to develop an affinity with other members of society and further consolidate their identities (Martin, 2009). From the perspective of resource allocation, culture is gradually allocated to persona through master identity and sub-cultures. In terms of affinity, individuals in alliance with others create subcultures with allocated semiotic resources and then establish a master identity, thereby enriching cultural systems (Martin, 2010). The two-way analysis of semiotic resource allocation and affinity attracts people’s attention to the language rules of “recognition” and “realization.” “Recognition” allows individuals to identify the uniqueness or similarity of a context and the legitimate coding orientation expected by the context while “realization” refers to the process where individuals produce languages and behaviors that meet context expectations (Lu, 2011). Social culture allocates semiotic resources to individuals according to their different identities, so individuals’ different coding orientations are formed. When an individual’s codes conform to the context, an alliance semantic system is formed (Zheng et al., 2021, 2022; Zheng and Yin, 2022). When an individual’s codes deviate from context expectations, the alliance is interrupted, and individuals tend to construct another identity. Therefore, if the construction of students’ individual potential deviates from the identification and recognition of self-identity and communicative identity in classroom discourse, it is easy to be assimilated by the other side’s discourse and depart from the original theme. The task of classroom teaching, on one hand, is to guide students to “identify” the different identities of textbook authors in the context of the situation in classroom discourse, deconstruct their coding orientations, and enhance their cognition. On the other hand, classroom teaching aims to help students learn to “realize” the coding orientation in accordance with their identities in specific contexts through acquired language strategies and transmit interpersonal meanings, thus improving individual CCCL meaning potential.

There are generally two viewpoints concerning cultural orientation in academic circles: essentialism and non-essentialism. The former represents the inherent culture and biological characteristics shared by a certain group while the latter integrates contextual influences and individual particularity into the culture from the perspective of development (Sun et al., 2020). From the aspect of development, people’s cultural experiences are affected by individual identity, covering race, gender, age, class, and religion, among others (Skelton and Allen, 1999). From the identity negotiation perspective (INP), identity is involved in eight fields, namely, culture, ethnicity, gender, personal, role, relational, facework, and symbolic interaction (Ting-Toomey, 2020). This kind of identity derives from the self-definition generated in the interactions between individuals and others and lays stress on the relationship between culture and individual identity. For this reason, speakers’ speech strategies were interpreted based on the eight fields of identity, and students were organized to construct their speech in line with their identities in class in this research. From INP, the eight identities can be divided into the ones (culture, ethnic, gender, and personal identities) in primary identities and those (role, relational, facework, and symbolic interaction identities) in situational identities. The former is relatively stable and exerts an impact throughout one’s life while the latter changes with different situations (Ting-Toomey, 2020).

### Identity Recognition in Classroom Discourse and Coding Realization

In this research, the interpersonal meaning constructed by the text author in a reading course for English majors was explored using *Reading Course in American* and *British News Publications* as the textbook. This textbook contains 40 pieces of American and British news divided into 10 sections, covering every aspect of social life, such as social groups, food, clothing, shelter, transportation, family and marriage, system, and concepts. In fact, the consciousness of authors and national cultural consciousness were implied in news regardless of its objective and fair image as a communication medium. In this research, the identity construction strategies of writers were analyzed with five pieces of news as the corpus, involving cultures, values, marriage, and others (Table 2) with the typical characteristics of cultural news.

**TABLE 2:** News corpus.

No.	Subject	Content
1	Why Haven’t You Gotten Deported?	American immigrants
2	The End of Men	Women status in America
3	No One Way to Keep Love in Bloom, Experts Say	View of marriage and love in America
4	NYC Ban Sparks Smoking War	Anti-smoking movement in America
5	Those Rugged Individuals	Individualism values in America

#### Identity Recognition in Primary Identities and Coding Realization

Cultural identity refers to “*the emotional significance that we attach to our sense of belonging or affiliation with the larger culture*” (Ting-Toomey, 2020), and commenting on values *via* the attitude system is the most straightforward appraisal method for the individual cultural identity, supplemented by the graduation and engagement systems. For instance, *Those Rugged Individualism* introduces the American individualism development history and characteristics; “sacred” and “coveted” were two positive words used in the opening “*No ideal may be held more sacred in America, or be more coveted by others, than the principle of individual freedom*” to explain the positive values of individualism in American society from two perspectives: “valuation” and the “reaction” of appreciation, and the graduation system was a supplement to illustrating its “supremacy.” Furthermore, the indicative mood of the monogloss system applied herein defined the position of individualism directly, leaving no negotiable space for readers. Therefore, the writer could be inferred as a supporter of individualism.

Historical changes have promoted the coexistence of various cultures in multinational countries, such as Asian Americans, African Americans, Latin Americans, and others in America. Individuals from different ethnic groups have their own original beliefs and identities that are influenced by families and communities, though they live in the same country. Specifically, in *Why Haven’t You Gotten Deported?*, the influences of illegal immigrant identity on the writer’s affects were described as below:

(1) Like many others, I kept my status a secret, passing myself off as a United States citizen right down to cultivating a homegrown accent. I went to college and became a journalist earning a staff job at the Washington Post. But the deception weighed on me.

From the perspective of attitude, Example (1) showed a negative affect of the writer as an illegal immigrant. The fact of feeling insecurity was conveyed indirectly to the readers by behaviors including “*kept my status a secret*,” “*passing off*,” “*the deception weighed on me*,” and so on. From the view of engagement, the linguistic strategy of dialogical contraction was applied without modality selection herein, leaving no dialogical space for the readers on the basis of a subjective statement of facts. In addition, the concession strategy was used in the last clause. The word “but” belonged to the category of heterogloss, expressing the meaning of anti-expectation: A lot of endeavors did not yet bring the writer a status and a pressure-free life as a legal United States citizen. In conclusion, each of the six clauses in Example (1) was a dialogical contraction, which revealed the identity characteristics of American illegal ethnic minorities straightforwardly to the readers with no negotiable space left.

As a result of the connection between gender identity and cultural identity, gender is positioned differently by the same culture in different periods and also so by different cultures in the same period. Students can realize their gender identity through classroom discourse (Banegas et al., 2020) and form gender-characterized discourses and behaviors. In articles describing the position changes of genders from a historical view, the engagement system is usually adopted to highlight their validity. In other words, the heterogloss system is generally used to provide examples for pure narratives, e.g., a French female writer’s description of women’s status was mentioned in *The End of Men* (Example 2).

(2) Women have killed themselves (or been killed) for failing to bear sons. The French feminist Simone de Beauvoir suggested that women so detested their own “feminine condition” that they regarded their newborn daughters with irritation and disgust.

In this paragraph, the three view angles of attitude, graduation, and engagement were used to judge the female status and recognize their identity. Overall, women’s equal status was denied by the negative words like “*kill*,” “*fail*,” “*detest*,” “*irritation*,” and “*disgust*,” and such process-description words like “kill” and “fail” were selected herein to promote the status changes caused by giving birth to a boy or not to a negative comment on the social sanction: women got killed or committed suicide if they did not bear sons. The word “*detest*” and the two nominalized emotional words “*irritation*” and “*disgust*” expressed the negative mentality produced from the fact of having a girl in the perspective of affect, and the three words had a very high semantic value, that is, their assigned degree of hatred and anger were higher than the ordinary words like “dislike,” “hate,” and “angry,” just echoing the monoglossically stated female status mentioned previously. Moreover, the paraphrase word “*suggest*” was used as the example of “acknowledgment” to indicate the female writer’s agreeable attitude toward this viewpoint. Owing to this external discourse, the descriptive demonstration of female identity and status got strengthened.

Individuals form their personal identity by observing the role needs around them according to their unique life experiences and personalities. Such identity which is related to individual emotions, knowledge, personal objectives, values, and motives is both influenced by the cultural identity and is attributed to the peculiarity of the personal identity (Ting-Toomey, 2020). Therefore, speakers select the coding orientation and construct their identity on basis of their own interests and needs. As seen in *NYC Ban Sparks Smoking War* (Examples 3–5), people of different identities had different opinions toward the ban on smoking, and then the identity-fitted utterance was constructed by different linguistic strategies.

(3) But the co-owner of Lotus, one of the hottest night spots in Manhattan, says he now spends a good part of his time fighting a law that prohibits lighting up in bars and pushes smokers onto the street.

(4) “If what I’m hearing is correct, this is having a devastating effect on the city’s economic recovery,” says Queens councilman Tony Avella, who says he reluctantly voted for the ban but thinks the council should revisit the issue.

(5) In a city of apartment dwellers, where people live above restaurants and pubs, some say long-standing tensions between businesses and residents have only risen since smokers were forced to congregate outside.

The stances of the businessman, the councilman, and the residents near the bar were shown in Examples (3–5), respectively. The radical word “*fight*” in Example (3) was used to demonstrate the passive attitude of the businessman, portraying his image of the discontent, and the phrase “*a good part of*” modified the time quantitatively, revealing the resistance from the businessman against the ban of smoking in consideration of the high time cost. From the view of the engagement system, the councilman in Example (4) kept their distance from the discourse resource using the “*if*” adverbial clause of concession to get rid of the responsibility for judging the severe crash in the economy. However, from the angle of the attitude system, the councilman held a negative attitude (“*reluctantly*”) toward the new ban on the stance of economic development. Herein, he made an objective response without personal emotions so as to fit his civil servant identity. Moreover, the conflict between the businessmen and residents narrated from a resident’s view in Example (5) was leveled up to a focus using “*long-standing*” and “*only*” from the graduation system, to illustrate the residents’ dissatisfaction without any inscribed attitude words though. In a word, the subjective attitude expressions in Examples (3) and (5) were in accordance with the ordinary citizen identity, while the objective stance of national economic development coincided with the councilman’s identity in Example (4).

To sum up, the basic role identity was related to resource distribution. The above four types of identity would not change with the context of the situation but keep stable. Accordingly, in the classroom teaching, students could be divided into several groups by the four primary identities after learning textual discourse strategies, based on which each group member took turns to contribute one sentence to construct the textual code on the basis of the group’s common identity. After the whole text was completed, representatives of each group reported to the class. In detail, four identity types, i.e., cultural identity, ethnic identity, gender identity, and personal identity, were included. Compared to the former three types, the personal identity was determined by one’s distinct experiences and personality, resulting in the impossibility of the same personal identity between two people. Consequently, different relational criteria could be adopted for grouping. In other words, students with similar objectives or personalities could voluntarily form a group to complete the text by coding in turn, and then, the group representative stated to the class their objective or personal identity of a particular personality.

#### Identity Recognition in Situational Identities and Coding Realization

Role identity in situational identities is connected to behaviors and values of cultural or national expectation, including social norms that restrict the behaviors of roles (Ting-Toomey, 2020). The role distribution contained in *Why Haven’t You Gotten Deported?* is an example of this. As seen in Example (6), the author first appreciated the pathway of becoming a legal United States citizen in an indicative mood, then closed queries with a negative polarity item “*doesn’t*,” and clarified the reliability of previous appraise, and used the word “*easiest*,” the superlative form to make a “self-mockery” on the illegal immigrant identity from the opposite side. The passive affect was throughout the text, telling the sadness of illegal immigrants, whereas such positive words (“*easiest*” and “*doesn’t*”) were chosen herein but for the description of native Americans. This kind of appreciation semantic value served as a foil to the pathetic illegal immigrants.

(6) The easiest way to become a United States citizen is to be born here—doesn’t matter who your parents are; you’re in.

In the process of learning cross-cultural coding orientation, the individual identity differences are caused by the differences in relational identity and facework identity in different sociocultural contexts, which are non-negligible. Family identity is one of the relational identities, and the initially formed “three outlooks” and social identity of individuals usually come from families. Practically, students could give comments on the family identity of Americans with respect to affect when analyzing the view of marriage in the classroom discourse. As shown in Example (7), a concession was made to stress the “*not lost faith*” and “*institution*” in the last clause, appraising the marriage positively from the angle of the security sense. Additionally, individuals can choose interpersonal relations and friendships according to the context and personal needs as they grow up. With a closer relationship, the discourses are less while the contents conveyed are more, and a looser relationship brings about more discourses but simpler meanings (Fang, 2014). Back to the facework identity, which is related to the respect between two communicating parties, it can be appraised from perspectives of the social esteem and social sanction. More specifically, comparing Example (1) with (6), the passive word “*weighed*” and the positive word “*easiest*” were contrasted to judge the fact that cultures provided infants of different births with different social acknowledgment and respect. The speaker made verbal responses in accordance with the particular context and decided whether to redeem reputation by linguistic strategies: (1) The engagement system could be applied to affirm the discourse and keep the identity relationship between two parties or to negate the other viewpoints and desert the existing relations. (2) The graduation system could be adopted to form a confrontational reading stance by strengthening the attitude or to form a subjective one and protect the facework identity as a result of the weakening mood. (7) Almost 90% of Americans marry at some point in their lives. An overwhelming number of those who get divorced marry a second time, meaning that, although they may have lost faith in a partner, they have not lost faith in the promise of the institution.

Linguistic coding orientation is one method of expressing individual identity, which is constructed by means of the discourse: How others talk to us, how others talk of us, and how we get closer to others by talking (Ivanič, 2006; Karam, 2018). Individuals have the power to choose the coding orientation in cultural system interaction, and the individual choices are displayed by the linguistic styles, namely, the individual signature, which manifests a relatively concrete personality and constructs the unique individual social identity (Lu, 2011). For instance, in this research, each student had different individual linguistic resources, such as the distinct manner of articulation, the volume of emotional expression, and the application of vocabulary types. Such characteristic linguistic knowledge and skills built students’ individual symbolic interaction identity.

As for the identity changing with the context of situations, the context-set teaching method could be used to model a context similar to that from the textbook and presume different types of identities at the coding realization stage of the classroom discourse. Then, students were grouped accordingly, and group members demonstrated their coding orientation based on the set context to each other. At last, the text of the individual was stated to the class.

## Conclusion

Inevitably, when cross-cultural literacy is placed under contemporary world cultural development, the cultivation of CCCL is involved and becomes the focus and difficulty for English majors in higher education. Empirical studies show that even “good students” with excellent English grades in NCEE lack critical literacy, and that is why the individual meaning potential of college students needs to be constructed and improved. According to the individuation of SFL, the individual coding orientation interacts with the identity construction. In other words, individuals choose codes based on the identity or discover the identity on the basis of codes, meaning that the identity-compliant linguistic coding is completed from a two-way perspective, the “recognition” and the “realization.” In this research, the eight identities of INP in cross-cultural communication were combined, and the appraisal system was adopted to “recognize” the discourse strategy of textbook authors. Moreover, the identity and cultural awareness implied in the discourse were critically analyzed to “realize” the construction and improvement of individual CCCL repertoire out of codes. This study closely follows the need of fostering students’ national consciousness under cultural globalization and is expected to provide a reference path for ideological and political teaching for English majors.

## Data Availability Statement

The original contributions presented in this study are included in the article/supplementary material, further inquiries can be directed to the corresponding author.

## Ethics Statement

The studies involving human participants were reviewed and approved by the National University of Defense Technology. Written informed consent to participate in this study was provided by the participants or their legal guardian/next of kin.

## Author Contributions

RZ and DL contributed to the conception and design of the study. DL organized the database. RZ performed the statistical analysis and wrote the first draft of the manuscript. Both authors contributed to the manuscript revision and read and approved the submitted version.

## Conflict of Interest

The authors declare that the research was conducted in the absence of any commercial or financial relationships that could be construed as a potential conflict of interest.

## Publisher’s Note

All claims expressed in this article are solely those of the authors and do not necessarily represent those of their affiliated organizations, or those of the publisher, the editors and the reviewers. Any product that may be evaluated in this article, or claim that may be made by its manufacturer, is not guaranteed or endorsed by the publisher.
